# The Global Trachoma Mapping Project

**Published:** 2014

**Authors:** Anthony W Solomon, Elizabeth Kurylo

**Affiliations:** Wellcome Trust Intermediate Clinical Fellow: Clinical Research Department, London School of Hygiene & Tropical Medicine, London, UK. anthony.solomon@lshtm.ac.uk; Communications Manager: International Trachoma Initiative, Decatur, USA. ekurylo@taskforce.org

In the largest disease-mapping project ever conducted, health workers trained by the Global Trachoma Mapping Project have already examined the eyelids of more than 1 million people in nearly 1,000 districts since December 2012. The data collected are being used to create the first truly complete global map of trachoma and trichiasis, due in March 2015.

Data on the prevalence of trachoma and trichiasis at country and district level are vital in order to plan public health interventions and to mobilise resources. The public health interventions are based on the SAFE strategy for trachoma elimination, a strategy endorsed by the World Health Organization (WHO). SAFE is short for Surgery, Antibiotics, Facial cleanliness and Environmental improvement. It involves offering individuals with trichiasis – the blinding consequence of trachoma – a surgical procedure to stop their lashes being in contact with the eyeball. In populations with active trachoma, SAFE also involves offering antibiotics, education (about facial cleanliness and other good hygiene practices) and environmental improvement to reduce the carriage and transmission of the bacterium that causes trachoma.

In order to plan surgical services adequately, it is useful to know the prevalence of trichiasis: that is, where there are people with trichiasis and approximately how many are affected. To plan antibiotic distribution, facial cleanliness, education and improvements in water and sanitation for trachoma control purposes, it is essential to know the prevalence of active trachoma. This information must be collected at health district level, because health districts (populations of 100,000–250,000) are the units in which SAFE is usually put into practice.

## Why is mapping needed?

Trachoma is thought to be endemic in 2,400 districts worldwide. By July 2012, district-level surveys had established prevalence estimates in less than half of these (1,115 districts). The rest, (1,285 districts) were merely suspected to be endemic, without sufficient information to either start interventions in those places or to confidently add them to the list of districts for which full-scale SAFE implementation did not seem necessary.

**Figure:**
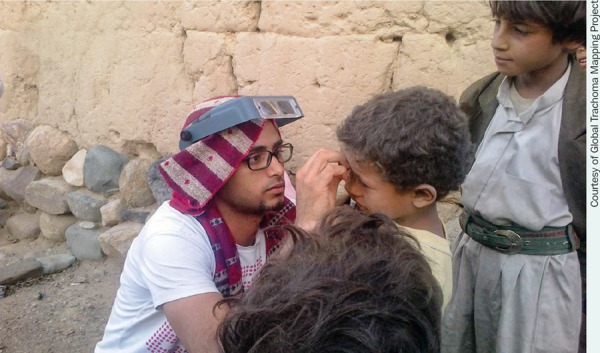
A health worker examines the eyelids of a child in Yemen.

In July 2012, therefore, Sightsavers, the International Trachoma Initiative (ITI) and the London School of Hygiene & Tropical Medicine, acting on behalf of the International Coalition for Trachoma Control, secured the £10.6 million Global Trachoma Mapping Project (GTMP) grant from the United Kingdom's Department for International Development. Following an intensive planning and piloting phase, mapping commenced in the Oromia Region of Ethiopia on 17 December 2012, soon followed by projects in other regions of Ethiopia and in other countries.

## How is the mapping conducted?

Each suspected endemic area is subdivided into ‘evaluation units’ comprising populations of 100,000–250,000 (these are generally equivalent to health districts). For the purposes of the mapping project, however, larger populations can be mapped as a single evaluation unit if trachoma is suspected to be highly and widely endemic.

In each project location, ministry of health staff are trained and certified by GTMP training teams. A population-based prevalence survey of more than 20 clusters (based on WHO guidelines) is then undertaken by those teams for each evaluation unit. Data are collected on water and sanitation at the household level, and on age, gender and the presence or absence of signs of trachoma at an individual level.

All data are collected electronically, using Android smartphones running the LINKS app, which has been developed and is maintained by the Task Force for Global Health, Atlanta, GA, USA. Data are geo-referenced using global positioning system coordinates; they are then transmitted to a high security server for cleaning and approval by the relevant ministry of health (using a site-specific password-protected web interface). Analysis of approved data is automatic, using pre-agreed algorithms, and prevalence categories are then displayed (with ministry of health agreement) on the web-based Global Atlas of Trachoma.^1^ Use of all of these elements of cutting-edge technology means that results can be ready for use for the population's benefit within days of fieldwork being completed. An additional advantage is that the results are considerably less prone to errors in data handling than the paper-based systems used in previous surveys.

The benefits of the GTMP approach have been recognised by ITI's Trachoma Expert Committee and by other agencies. For example, two non-governmental organisations (FHI360 and RTI International), both funded by the United States Agency for International Development, are working with the GTMP to conduct trachoma surveys in countries whose trachoma programmes they support.

## Progress thus far

By the beginning of March 2014, the GTMP was working with 18 implementing organisations in more than 30 countries, and some of the 21 million ‘bits’ of GTMP data had already been put to use to approve the deployment of Pfizer-donated azithromycin for mass antibiotic distribution. The project will continue until March 2015, paving the way for the launch of SAFE interventions – wherever required – to reach the 2020 target of eliminating trachoma as a public health problem.
